# Reducing the Cost of Implementing Filters in LoRa Devices

**DOI:** 10.3390/s19184037

**Published:** 2019-09-19

**Authors:** Shania Stewart, Ha H. Nguyen, Robert Barton, Jerome Henry

**Affiliations:** 1 Department of Electrical and Computer Engineering, University of Saskatchewan, 57 Campus Dr., Saskatoon, SK S7N 5A9, Canada; ha.nguyen@usask.ca; 2 Cisco Systems Canada, 2123-595 Burrard St., Vancouver, BC V7X 1L7, Canada; robbarto@cisco.com; 3 Cisco Systems, Research Triangle Park, 7100-8 Kit Creek Road, RTP, NC 27560, USA; jerhenry@cisco.com

**Keywords:** LoRa, chirp spread spectrum (CSS), Internet of Things (IoT), pulse shaping filter, multiplier-less filters, sample quantization

## Abstract

This paper presents two methods to optimize LoRa (Low-Power Long-Range) devices so that implementing multiplier-less pulse shaping filters is more economical. Basic chirp waveforms can be generated more efficiently using the method of chirp segmentation so that only a quarter of the samples needs to be stored in the ROM. Quantization can also be applied to the basic chirp samples in order to reduce the number of unique input values to the filter, which in turn reduces the size of the lookup table for multiplier-less filter implementation. Various tests were performed on a simulated LoRa system in order to evaluate the impact of the quantization error on the system performance. By examining the occupied bandwidth, fast Fourier transform used for symbol demodulation, and bit-error rates, it is shown that even performing a high level of quantization does not cause significant performance degradation. Therefore, the memory requirements of LoRa devices can be significantly reduced by using the methods of chirp segmentation and quantization so as to improve the feasibility of implementing multiplier-less filters in LoRa devices.

## 1. Introduction

The LoRa (Low-Power Long-Range) modulation technique is an excellent solution for many internet-of-things (IoT) applications due to its excellent energy consumption, link robustness and long-range capabilities at the expense of low bit rates [1,2]. LoRa uses a modified form of chirp spread spectrum (CSS) modulation, wherein the carrier frequency of a sinusoid is linearly varied across a specific bandwidth. This results in a set of signals known as chirps [3], which are distinguishable by their starting frequencies.

The behavior of a LoRa chirp is controlled by both the spreading factor, SF, and the bandwidth parameter, BW. The spreading factor is an integer value, typically ranging from 6 to 12, while the specified bandwidth can be chosen from values in the range of 7.8 to 500 kHz [4,5]. Each chirp (or symbol) is encoded with SF bits, which means that there are $M=2^{SF}$ possible symbol values, where *M* is the modulation order [6]. The instantaneous frequency of a chirp linearly increases or decreases across the bandwidth specified by BW over the symbol duration [3]. The tradeoff between the range capabilities and the nominal bit rate depends on SF and BW. For instance, high SF and low BW allow for higher receiver sensitivity, but at a lower bit rate, whereas low SF and high BW lead to reduced receiver sensitivity, but a higher bit rate.

As the demand for long-range, low-power IoT devices increases, so does the need to improve the spectral efficiency of these devices’ transmission. One promising solution is to implement a set of pulse shaping and matched square-root raised cosine (SRRC) filters in LoRa transmitters and receivers, respectively. The use of these filters can significantly reduce the bandwidth containing 99% of the total mean signal power while also reducing the out-of-band emissions created by LoRa devices [6,7,8]. The increase in spectral efficiency allows us to accommodate a larger number of IoT devices.

The challenge is that since LoRa devices are characterized by their low complexity, it is more difficult to justify the added resources required for filtering, especially when longer filters are required (which is often the case for LoRa devices with lower bandwidth settings [8]). Since the cost of implementing multipliers in hardware can prove significant, a preferred solution should eliminate the need for multiplications altogether. As such, the main objective of this paper is to investigate the feasibility of implementing a “multiplier-less” pulse shaping filter in a LoRa transmitter.

Replacing hardware multipliers in a pulse-shaping filter can be done with a look-up table (LUT), provided there is a finite number of input sample values to the filter that are known. Instead of multiplying each incoming sample by a filter coefficient using a hardware multiplier, the result of every possible multiplication can be precalculated and stored in the LUT. Then, the LUT can output the correct product of the required multiplication based on the associated input sample value. The size of the LUT depends on both the number of unique input sample values and the filter length. In such an implementation, the complexity of the filter is measured by the cost of the memory instead of hardware multipliers.

The problem with LoRa is that since discrete-time LoRa chirps are made up of $M=2^{SF}$ samples, the multiplier-less filter must be able to accommodate the *M* possible input values for each chirp waveform. Furthermore, implementing multiple SF settings and ensuring the continuous phase of modulated chirp waveforms exponentially increase the already large number of unique filter inputs. While many of the chirp sample values are repeated among spreading factors and/or symbol values, the memory requirement of the LUT is still significant. The LoRa end-devices are particularly constrained by the additional memory requirements as these devices have a greater need for low energy consumption and few complex operations than the LoRa gateway.

In this paper, two methods of optimizing LoRa transmitters are proposed in order to reduce the complexity of filtering. First, waveform segmentation is used to generate an entire basic LoRa chirp waveform from only a portion of the total number of chirp samples in order to reduce the size of the chirp generation ROM. While this does not directly impact the LUT size, it reduces the overall memory requirement. This method is inspired by the CSS transceiver design presented in [9] and it has been adapted for LoRa.

The second method involves quantizing the LoRa chirp samples to a significant degree so as to reduce the number of unique input values to the multiplier-less filter, which helps to reduce the LUT size. While chirp segmentation does not add any error, quantization adds some rounding errors to the quantized chirp signals. It is important to ensure that the desired sample reduction can be achieved without significant performance degradation.

The feasibility of implementing a multiplier-less SRRC pulse shaping filter in a LoRa transmitter will be evaluated in terms of the tradeoff between the potential sample reduction and impact of quantization noise on the performance. In order to quantify the effectiveness of the sample reduction, the number of samples required to form LoRa chirp waveforms and subsequent filtered signals shall be compared to that of an unoptimized LoRa device. The degree of sample reduction, therefore, depends on which spreading factors are supported, the length of the pulse shaping filter, if chirp segmentation is used, and the quantization step size (if any).

Finally, the performance of the system will be evaluated for LoRa signals with various degrees of quantization. A LoRa communication system is simulated in Matlab, and the performance is evaluated in terms of the occupied bandwidth (OBW) of transmitted LoRa signals, the output of the fast Fourier transform (FFT) performed for symbol demodulation, and the bit-error-rate (BER). The goal is to find appropriate levels of quantization in order to significantly reduce the overall memory requirement while maintaining excellent performance.

## 2. Sample Reduction Methods

**Figure 1:**
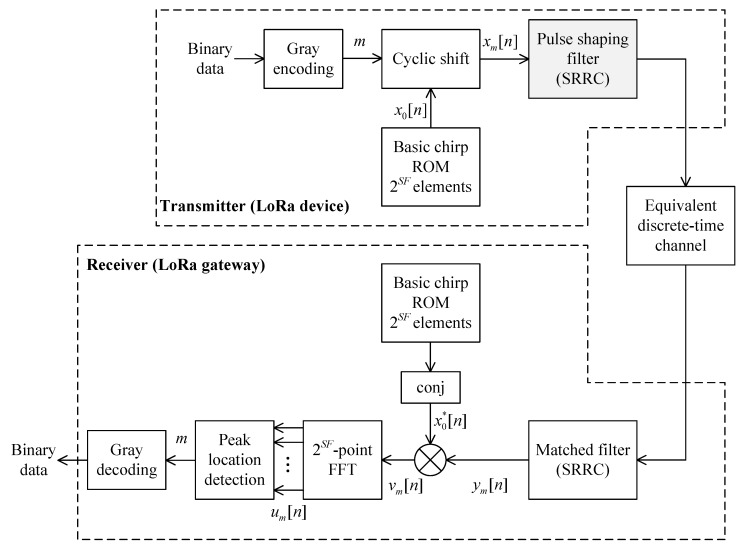
Block diagram of a LoRa (Low-Power Long-Range) system implementing pulse shaping and matched filters.

Figure 1 illustrates a block diagram of a LoRa system that implements a pulse shaping filter at the transmitter and a matched filter at the receiver. While the performance benefits brought by implementing these filters are demonstrated in detail in [6,8], this paper focuses on reducing the complexity of implementing the pulse shaping filter in a LoRa end device’s transmitter.

**Figure 2:**
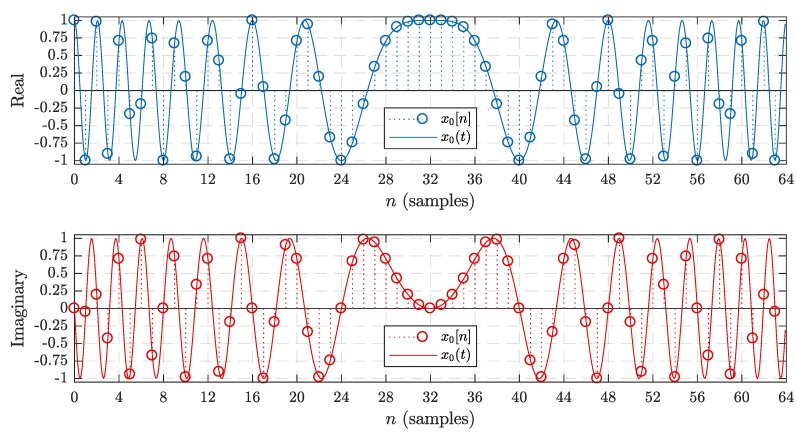
Example of discrete-time and continuous-time basic chirp waveforms with SF=6 and BW=125 kHz.

Basic LoRa chirp waveforms are used as the basis for the LoRa modulation technique. They are used in both the preamble and payload of transmitted LoRa packets. The expression for a continuous-time basic LoRa chirp waveform is shown in (1), where $T_{\mathrm{sym}}$ is the symbol duration in seconds and μ is the chirp rate in Hz/second. The continuous-time chirp waveform is then sampled at a rate of $F_{s}=\frac{1}{T_{s}}=BW$ for digital implementation [6]. The expression for a discrete-time basic LoRa chirp is given below in (2), where $t=nT_{s}=n/BW$. As an example, Figure 2 plots the real and imaginary components of both $x_{0}\left[n\right]$ and $x_{0}\left(t\right)$ with SF=6 and BW=125 kHz.

(1) $$\begin{array}{cc} x_{0}\left(t\right) & =\exp\left[j2\pi \left(\frac{\mu t}{2}-\frac{BW}{2}\right)t\right],\;0\leq t\leq T_{\mathrm{sym}} \end{array}$$

(2) $$\begin{array}{cc} x_{0}\left[n\right] & =\exp\left[j2\pi \left(\frac{n^{2}}{2M}-\frac{n}{2}\right)\right],\;n=0,1,\ldots ,M-1. \end{array}$$

LoRa symbols are modulated by cyclically shifting the basic chirp waveform by the symbol value, *m*, as shown in (3).

(3) $$\begin{array}{cc} x_{m}\left[n\right] & =x_{0}\left[n+m\right]=\exp\left[j2\pi \left(\frac{{\left(n+m\right)}^{2}}{2M}-\frac{\left(n+m\right)}{2}\right)\right]\\ \; & =\exp\left[j2\pi \left(\frac{n^{2}}{2M}-\frac{n}{2}+\frac{m^{2}}{2M}-\frac{m}{2}+\frac{nm}{M}\right)\right]=x_{0}\left[n\right]x_{0}\left[m\right]\exp\left[j2\pi \left(\frac{nm}{M}\right)\right]. \end{array}$$

Furthermore, in order to maintain the phase continuity between subsequent chirps, each modulated chirp waveform obtained from (3) is multiplied by the complex conjugate of its first sample, $x_{m}^{*}\left[0\right]$. This causes the instantaneous phase of the chirp to be zero at both the beginning and end of the symbol duration, rather than causing sharp phase discontinuities between consecutive modulated chirps [3,6]. Performing phase correction changes the modulated chirp waveform expression given by (3) to that of (4).

(4) $${\hat{x}}_{m}\left[n\right]=x_{m}\left[n\right]x_{m}^{*}\left[0\right]=\exp\left[j2\pi \left(\frac{n^{2}}{2M}-\frac{n}{2}+\frac{nm}{M}\right)\right]=x_{0}\left[n\right]\exp\left[j2\pi \left(\frac{nm}{M}\right)\right].$$

While performing the complex multiplication will not affect the number of samples required for chirp generation, it will drastically affect the number of unique filter inputs. However, as in the case of basic chirp samples, it turns out that many phase-corrected sample values are shared among multiple SF settings, as well as modulated chirp waveforms associated with other symbols.

In terms of implementation, the real and imaginary components are considered separately as they correspond to the I and Q channels in a practical system. However, since the magnitude of $x_{0}\left[n\right]$ at each sample index is always equal to 1, the sequence of sample values for the I and Q channels are subject to the same patterns. Therefore, the properties discussed in this paper that are used to simplify the chirp generation shall apply to both the real and imaginary samples of $x_{0}\left[n\right]$. Both components also contain the same sample values, albeit the signs and sample indices may differ.

With that in mind, the following subsections detail the proposed methods and the resulting sample reduction compared to a standard LoRa device. It is also important to note that while basic chirp samples obtained from (2) are used for chirp generation, the LUT must account for each possible phase-corrected modulated chirp sample obtained from (4).

### 2.1. Chirp Waveform Segmentation

It is perhaps more intuitive to begin by examining the inherent symmetry in the sequence of $M=2^{SF}$ basic chirp samples that make up the waveform. For instance, consider the basic chirp waveform shown in Figure 2 once again. Both components appear to exhibit a symmetry about the midpoint located at $n=\frac{M}{2}$, which is n=32 in this case.

While it may not be obvious from Figure 2, there are several other patterns present in the sequence of chirp sample values as well. A close inspection reveals that a basic chirp waveform can be divided into four segments, each containing $\frac{M}{4}$ samples according to (5) and (6), where *k* is an integer representing the segment number. More importantly, each of these segments contains identical sample values, but they differ with predictable patterns of opposing signs and/or sample order.

(5) $$\begin{array}{c} x_{0,k}\left[n\right]=x_{0}\left[n+\frac{(k-1)M}{4}\right],\;n=0,1,\ldots ,\frac{M}{4}-1;\;k=\left\{1,2,3,4\right\} \end{array}$$

(6) $$\begin{array}{c} x_{0}\left[n\right]=\left\{\begin{array}{c} \begin{array}{cc} \;x_{0,1}\left[n\right], & \;0\leq n\leq \frac{M}{4}-1\\ \;x_{0,2}\left[n\right], & \;\frac{M}{4}\leq n\leq \frac{M}{2}-1\\ \;x_{0,3}\left[n\right], & \;\frac{M}{2}\leq n\leq \frac{3M}{4}-1\\ \;x_{0,4}\left[n\right], & \;\frac{3M}{4}\leq n\leq M-1 \end{array} \end{array}\right. \end{array}$$

Using (5) with (2) gives the exponential form of $x_{0,k}\left[n\right]$ shown in (7). Substituting each value of *k* into (7) gives the individual waveform segment expressions shown in (8).

(7) $$\begin{array}{c} x_{0,k}\left[n\right]=\exp\left[j2\pi \left(\frac{n^{2}}{2M}-\frac{n}{2}+\frac{(k-1)n}{4}\right)\right]=x_{0}\left[n\right]\exp\left[j\pi \left(\frac{(k-1)n}{2}\right)\right] \end{array}$$

(8) $$\begin{array}{c} x_{0,k}\left[n\right]=\left\{\begin{array}{cc} \;\exp\left[j2\pi \left(\frac{n^{2}}{2M}-\frac{n}{2}\right)\right], & k=1\\ \;\exp\left[j2\pi \left(\frac{n^{2}}{2M}-\frac{n}{2}\right)\right]\exp\left(j\pi \frac{n}{2}\right), & k=2\\ \;\exp\left[j2\pi \left(\frac{n^{2}}{2M}-\frac{n}{2}\right)\right]\exp\left(j\pi n\right), & k=3\\ \;\exp\left[j2\pi \left(\frac{n^{2}}{2M}-\frac{n}{2}\right)\right]\exp\left(j\pi \frac{3n}{2}\right), & k=4 \end{array}\right.. \end{array}$$

As an example, consider the basic chirp sample values of $x_{0,k}\left[n\right]$ by segments for SF=6 shown in Table 1. It is important to note that while the analysis below refers to this specific set of data, the following relationships between segments hold for all SF settings.

The first segment, $x_{0,1}\left[n\right]$, can be manipulated in order to obtain the remaining three segments with relatively simple operations. First, consider segments 1 and 3. It is obvious from Table 1 that every odd sample of $x_{0,3}\left[n\right]$ has the opposite sign of $x_{0,1}\left[n\right]$ at the same sample index value, *n*. This relationship can be obtained mathematically by comparing the expressions for k=1 and k=3 in (8) as shown below.

$$x_{0,3}\left[n\right]=\exp\left[j2\pi \left(\frac{n^{2}}{2M}-\frac{n}{2}\right)\right]\exp\left(j\pi n\right)=x_{0,1}\left[n\right]\exp\left(j\pi n\right).$$

Next, consider the relationship between segments 1 and 4. It is simple to see that $x_{0,4}\left[n\right]$ is a reverse indexed copy of $x_{0,1}\left[n\right]$. This can be proven by first finding $x_{0,1}\left[\frac{M}{4}-n\right]$ as shown below, and then comparing the resulting expression to that of $x_{0,4}\left[n\right]$.

$$\begin{array}{cc} x_{0,1}\left[\frac{M}{4}-n\right] & =\exp\left[j2\pi \left(\frac{{\left(\frac{M}{4}-n\right)}^{2}}{2M}-\frac{\frac{M}{4}-n}{2}\right)\right]=\exp\left[j2\pi \left(\frac{n^{2}}{2M}-\frac{n}{4}+\frac{n}{2}-\frac{3M}{16}\right)\right]\\ \; & =\exp\left[j2\pi \left(\frac{n^{2}}{2M}-\frac{n}{2}\right)\right]\exp\left(j\pi \frac{3n}{2}\right)\underset{=1\;\mathrm{for}\;\mathrm{all}\;M}{\underset{︸}{\exp\left(-j\pi \frac{3M}{8}\right)}}\\ \; & =x_{0,1}\left[n\right]\exp\left(j\pi \frac{3n}{2}\right)=x_{0,4}\left[n\right]. \end{array}$$

Lastly, $x_{0,2}\left[n\right]$ is a reverse indexed copy of $x_{0,1}\left[n\right]$ with opposing signs at every odd value of *n*. This is confirmed by the following comparison between the expressions for $x_{0,1}\left[\frac{M}{4}-n\right]$ and $x_{0,2}\left[n\right]$.

$$x_{0,1}\left[\frac{M}{4}-n\right]=x_{0,1}\left[n\right]\exp\left(j\pi \frac{3n}{2}\right)=x_{0,1}\left[n\right]\exp\left(j\pi \frac{n}{2}\right)\exp\left(j\pi n\right)=x_{0,2}\left[n\right]\exp\left(j\pi n\right).$$

In summary, $x_{0,2}\left[n\right]$, $x_{0,3}\left[n\right]$, and $x_{0,4}\left[n\right]$ can be found from $x_{0,1}\left[n\right]$ using (9)–(11), respectively. These relationships can be used to generate the *M* basic chirp waveform samples from only the first $\frac{M}{4}$ samples in $x_{0,1}\left[n\right]$. As a result, the number of samples stored in the ROM can be reduced from a total of 2M to $\frac{M}{2}$ real and imaginary samples without introducing any error.

(9) $$\begin{array}{cc} x_{0,2}\left[n\right] & =\left\{\begin{array}{c} \begin{array}{cc} -x_{0,1}\left[\frac{M}{4}-n\right], & \;\mathrm{for}\;n\;\mathrm{odd}\\ x_{0,1}\left[\frac{M}{4}-n\right], & \;\mathrm{otherwise} \end{array} \end{array}\right. \end{array}$$

(10) x0,3[n]=−x0,1[n],M4−nfornoddx0,1[n],M4−notherwise

(11) $$\begin{array}{cc} x_{0,4}\left[n\right] & =x_{0,1}\left[\frac{M}{4}-n\right]. \end{array}$$

In order to quantify the impact of chirp segmentation on the complexity of a practical system, the actual number of real and imaginary samples contained in the ROM and LUT must be considered. Let $N_{\mathrm{gen}}$ represent the number of samples required for chirp generation, while $N_{\mathrm{in}}$ represents the number of unique samples at the input of the pulse shaping filter. The calculated values of $N_{\mathrm{gen}}$ and $N_{\mathrm{in}}$ are shown in Table 2 for a LoRa system using the chirp segmentation method. It should be pointed out that “all” means the support of spreading factors ranging from 6 to 12 in the scope of the study. Here, $N_{\mathrm{gen}}$ is calculated as $\frac{M}{2}$, while $N_{\mathrm{in}}$ is found by counting the number of unique sample values given by (4) for each possible symbol value. Furthermore, $N_{\mathrm{in}}$ is counted based on the absolute (unsigned) value of each sample value. This is because the sign of the input samples to the filter can be easily detected and the sign of the corresponding LUT output can be corrected accordingly (by taking the two’s complement), if necessary.

The number of samples required in the filter LUT depends on both the number of filter coefficients ($N_{\mathrm{filt}}$) and $N_{\mathrm{in}}$. The output values of the LUT are found by multiplying each filter coefficient by each unsigned filter input value. Since the filter coefficients are symmetric about the midpoint of the filter, the number of stored multiplications can be reduced to just over half the number of filter coefficients instead. As a result, the total number of samples that must be stored in the transmitter for each spreading factor can be found using (12). It should be noted that the filter input value of zero included in $N_{\mathrm{in}}$ can be disregarded since the output of the coefficient multiplication(s) will simply be zero as well.

(12) $$N_{\mathrm{TX}}=\frac{N_{\mathrm{filt}}+1}{2}\left(N_{\mathrm{in}}-1\right)+N_{\mathrm{gen}}.$$

As an example, Table 3 displays the total numbers of samples required for three different systems calculated with (12). This example considers a standard LoRa device that does not use chirp segmentation or a multiplier-less filter ($N_{\mathrm{filt}}=0$), and two devices using chirp segmentation with length-17 and 81 multiplier-less SRRC filters, respectively. The filter lengths were selected based on their ability to reduce the occupied bandwidth of LoRa signals for different BW settings [8]. When supporting individual spreading factors, the length-17 filter requires almost double the number of stored samples compared to the standard system. However, when supporting multiple spreading factors, the difference is not as substantial. Furthermore, if the standard device implements filtering with hardware multipliers, it will require the use of at least $\frac{N_{\mathrm{filt}}+1}{2}$ multipliers for the filter in addition to the samples provided in Table 3.

In this regard, chirp segmentation improves the feasibility of implementing the length-17 filter without significant resource usage. For accommodating longer filters, the use of chirp segmentation alone does not provide a significant reduction in complexity due to the large number of samples. However, these results were obtained by modelling the system with a very small quantization step size (i.e., Matlab precision) in order to exactly represent the theoretical response. If the quantization step size is increased, it is possible to reduce the number of unique filter input values in order to accommodate the use of longer filters. This is discussed further in the next section.

### 2.2. Quantization

In order to implement a practical LoRa system, some level of quantization is necessary to represent the LoRa chirp signals. Since the chirp sample values are normally between ±1, two integer bits are needed to represent a signed chirp signal. Thus, only the number of fractional bits can be varied and investigated. Let *B* represent the number of fraction bits used for the system such that the uniform quantization step size is $Q=2^{-B}$.

Assuming the use of chirp segmentation, the values of $N_{\mathrm{gen}}$ will be those found in Table 2 as before. However, $N_{\mathrm{in}}$ depends on the quantization factor, i.e., the number of fraction bits, *B*. Table 4 contains the values of $N_{\mathrm{in}}$ found for five different values of *B*, namely 2, 4, 6, 8, and 10 bits.

Once again, the total number of samples required to implement the LoRa transmitter for each quantization factor can be found from (12). The calculated values of $N_{\mathrm{TX}}$ for LoRa devices utilizing chirp segmentation and quantization are shown in Table 5 and Table 6 with filter lengths of 17 and 81 taps, respectively. Note that the results for the standard system do not change with quantization.

By comparing the results in Table 5 and Table 6 with that of Table 3, it is clear that quantization provides a more significant reduction in stored samples than using chirp segmentation alone. In fact, using chirp segmentation and quantization not only can match, but improve upon the results obtained for a standard system that does not use multiplier-less filtering. Representing the real and imaginary chirp samples with 8-bit fractional precision for both cases of filters would be an appropriate solution in this regard. Not only is there a significant sample reduction from the standard case, but no hardware multipliers would be required. The main concern with quantizing the LoRa chirp signals is the potential impact on the decoding performance. The performance results and comparison presented in the next section shall remove this concern.

## 3. Simulation Results

The performance of different LoRa communication systems with additive white Gaussian noise (AWGN) channels was evaluated in Matlab. The pulse shaping and matched SRRC filters both have an upsampling/downsampling factor of L=4, roll-off factor of β=0.10, and 81 coefficients. In terms of quantization, fractional bit-precisions of 2, 4, 6, 8, and 10 were studied to quantize both the basic chirp samples generated at the transmitter and the conjugate basic chirp samples used for dechirping in the receiver. Additionally, a system was also tested without quantization to act as a reference.

### 3.1. Occupied Bandwidth

The occupied bandwidth measurements were taken on a Keysight N9030A Spectrum Analyzer [10] using the 99% power bandwidth method detailed in Section 6.9.3 of the ANSI standard for Compliance Testing of Unlicensed Wireless Devices [11]. The ANSI standard was referenced alongside the measurement guidelines for LoRa devices provided by Semtech [12] in order to comply with FCC regulations as well.

Each set of transmitted signals consists of 10 preamble symbols and 250 modulated chirp symbols. The signals were generated in Matlab and then sent to a Keysight N5182B Signal Generator [13]. The central carrier frequency was set to 915 MHz and the transmit power was set to 0 dBm. Since the upsampling factor is equal to 4, the sampling frequency of both the signal generator and spectrum analyzer was set to $F_{s}=L\times BW=4BW$. Measurements were obtained with the occupied BW mode using the peak detector and max hold traces on the spectrum analyzer. The values were recorded once the traces had stabilized after sweeping across over 350 points. The spectrum analyzer settings, which are summarized in Table 7, were varied with the LoRa bandwidth to comply with the standard [11].

The LoRa spreading factor was set to 10, while the specified bandwidth was set to 125, 250, and 500 kHz. The measured OBW results can be found in Table 8 for each tested bandwidth setting and fractional-bit precision. It is clear from these results that most tested levels of quantization do not impact the measured OBW. Even in the worst case, the difference is only a little over 100 Hz.

**Figure 3:**
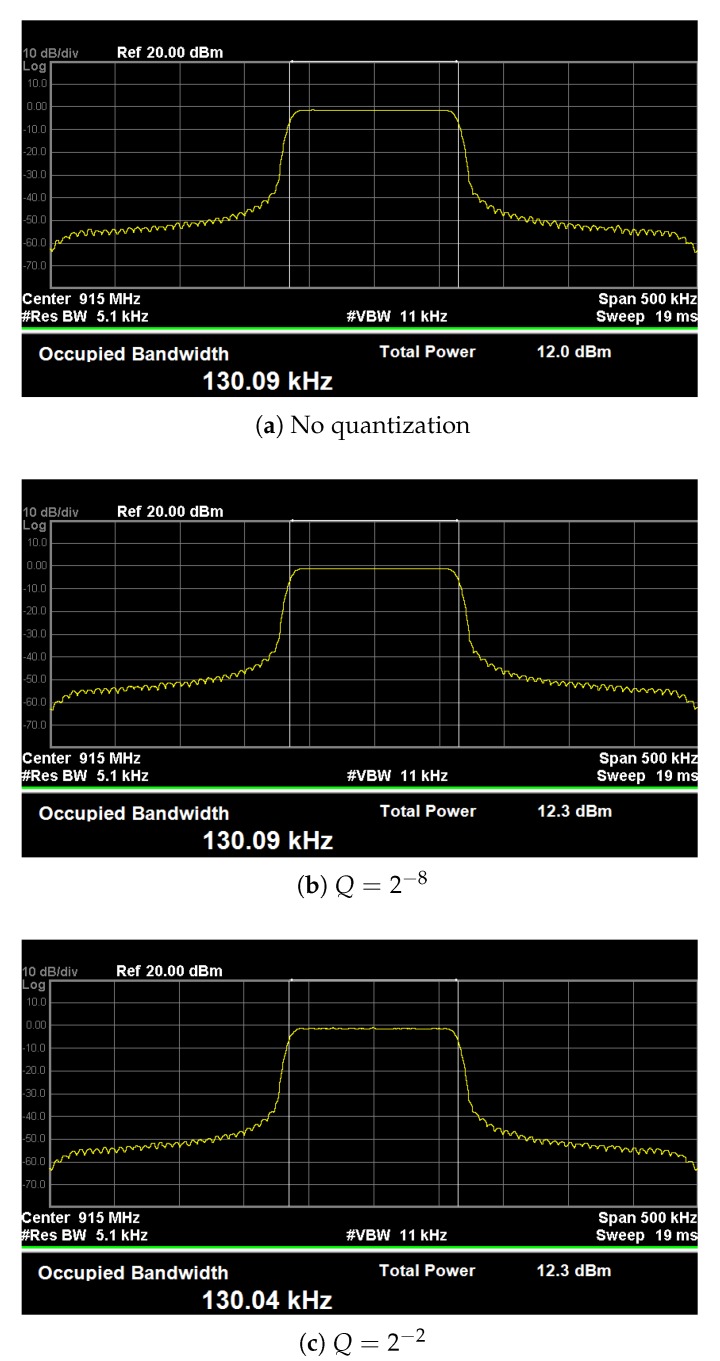
Occupied bandwidth measurements of filtered transmit signals with and without quantization when SF=10 and BW=125 kHz.

Additionally, consider sample screenshots taken from the spectrum analyzer shown in Figure 3. There is some noticeable distortion in the passband of Figure 3c and a small change in OBW due to the quantization error. However, there are no noticeable differences between the spectra shown in Figure 3a,b, even though the later is for signals that were quantized to 8 fractional bits. While the example presents results for SF=10, the measured OBWs for devices with different spreading factors and bandwidth settings also showed minor differences at high levels of quantization. In general, quantizing the chirp samples in the transmitter to a moderate degree does not appear to significantly distort the OBW measurements or the shape of the signal spectra.

### 3.2. FFT and Signal Spectrograms

As illustrated in Figure 1, LoRa symbol demodulation begins with a process known as dechirping [6]. Each received LoRa chirp waveform is multiplied by the complex conjugate of a basic upchirp having the same SF and BW. The product is a pure sinusoid whose frequency corresponds to the frequency offset associated with the modulated symbol value, *m* [3,6]. The frequency of the dechirped signal is then found by taking the *M*-point FFT and detecting which frequency bin contains the maximum energy. The index of the detected frequency bin is the LoRa symbol value.

**Figure 4:**
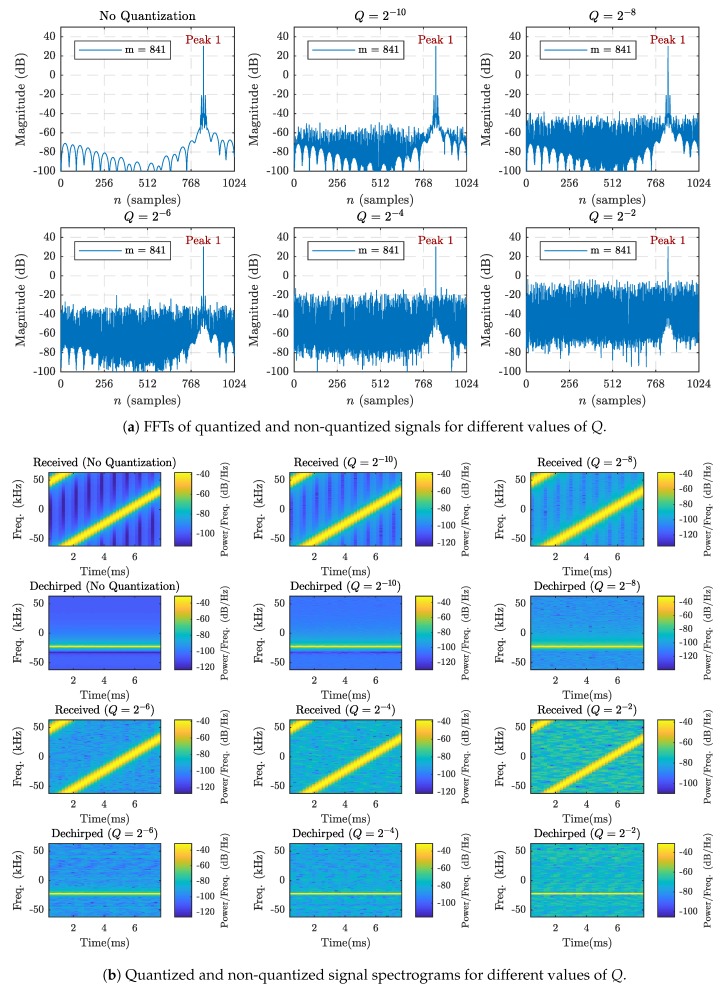
Fast Fourier transform (FFT) and spectrograms for filtered modulated chirps with and without quantization when SF=10, BW=125 kHz, and m=841.

Since decoding the proper symbol value depends on accurate peak detection, it is important to ensure that the peak associated with the symbol value is clearly distinguishable from that of the quantization noise. Figure 4 shows the *M*-point FFTs and spectrograms associated with quantized and non-quantized LoRa signals. Each tested LoRa signal corresponds to a symbol value of m=841, spreading factor of 10, and bandwidth of 125 kHz.

While there is a clear increase in the noise level when the quantization step size increases, the desired FFT peaks, and frequency ramps remain clearly visible for each case. Even with only 2-bit fractional precision, the quantization noise is well over 20 dB below the peak of the desired frequency bin. Furthermore, the spectrograms for the cases where B=8 and B=10 show hardly any noticeable distortion from the non-quantized case. It is clear that even with significant amounts of quantization noise, information symbols can be decoded properly provided there is no severe noise introduced by the channel.

### 3.3. Bit-Error Rate

**Figure 5:**
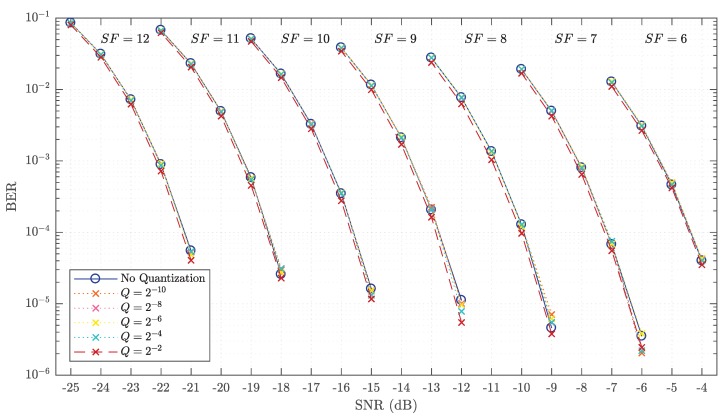
Bit-error-rate (BER) results for quantized and non-quantized signals with BW=125 kHz and SF ranging from 6 to 12.

The BER tests were performed for filtered LoRa signals with and without quantization to see how they would perform under the effects of noise in an AWGN channel. The bandwidth was set to a fixed value of 125 kHz, while the spreading factor was varied from 6 to 12 for all tests. The BER results for each case are shown in Figure 5. The BER was tested at each desired signal-to-noise ratio (SNR) level by transmitting 175,000 data bursts containing 10 LoRa symbols each. The transmit signal is normalized to have unit power and hence the noise power is calculated based on the desired SNR level as $P_{\mathrm{noise}}=10^{\frac{-\mathrm{SNR}}{10}}$.

The results for the non-quantized case can be corroborated by the simulated BER measurements of typical LoRa systems given in both [6,14]. It is evident that the results for most of the quantized cases match the non-quantized results, with the exception of the case with two-bit fractional precision. As such, it can be concluded that quantizing LoRa chirp signals to a certain degree does not affect the symbol decoding capabilities of LoRa devices in the presence of noise from an AWGN channel.

## 4. Conclusions

Two methods were presented to reduce the complexity of implementing multiplier-less SRRC pulse shaping filters in LoRa transmitters. These methods focus on reducing the required number of samples in the ROM used to generate basic chirp signals, as well as those required for the multiplier-less filter LUT. Chirp segmentation can be used to generate the entire basic LoRa chirp waveform from only a quarter of its samples without adding any additional error to the signal. Quantization can also be used to exponentially decrease the number of unique samples at the input to the multiplier-less pulse shaping filter at the cost of introducing small errors to the transmitted signal.

Using both methods allows for a reduction in the number of stored samples so as to not only match, but also improve upon the results obtained from a standard LoRa device that does not contain a multiplier-less filter. For example, a system using 10-bit fractional precision and a length-17 multiplier-less pulse shaping filter requires fewer samples to be stored in memory compared to a standard LoRa system when supporting spreading factors 6 to 12. Even a device with a length-81 filter requires fewer stored samples than a standard device by quantizing the LoRa chirp samples to 8 fractional bits.

Furthermore, it was shown that moderate levels of quantization do not hinder the decoding performance of LoRa devices, even under harsh channel conditions. Therefore, the quantization factor can be chosen based on the complexity requirements of the system. For example, devices intended for long-range communication require larger spreading factors and, as a result, a higher quantization factor to compensate for the added complexity. In conclusion, using the proposed sample reduction methods can aid in further alleviating the complexity concerns associated with implementing SRRC filters in LoRa devices.

## Figures and Tables

**Table 1:** Discrete-time basic chirp samples by segments of $x_{0}\left[n\right]$ for SF=6.

$x_{0,1}\left[n\right]=x_{0}\left[n\right]$			$x_{0,2}\left[n\right]=x_{0}\left[n+16\right]$			$x_{0,3}\left[n\right]=x_{0}\left[n+32\right]$			$x_{0,4}\left[n\right]=x_{0}\left[n+48\right]$		
n	**Real**	**Imag.**	n	**Real**	**Imag.**	n	**Real**	**Imag.**	n	**Real**	**Imag.**
0	1	0	0	1	0	0	1	0	0	1	0
1	−0.9988	−0.0491	1	0.0491	−0.9988	1	0.9988	0.0491	1	−0.0491	0.9988
2	0.9808	0.1951	2	−0.9808	−0.1951	2	0.9808	0.1951	2	−0.9808	−0.1951
3	−0.9040	−0.4276	3	−0.4276	0.9040	3	0.9040	0.4276	3	0.4276	−0.9040
4	0.7071	0.7071	4	0.7071	0.7071	4	0.7071	0.7071	4	0.7071	0.7071
5	−0.3369	−0.9415	5	0.9415	−0.3369	5	0.3369	0.9415	5	−0.9415	0.3369
6	−0.1951	0.9808	6	0.1951	−0.9808	6	−0.1951	0.9808	6	0.1951	−0.9808
7	0.7410	−0.6716	7	−0.6716	−0.7410	7	−0.7410	0.6716	7	0.6716	0.7410
8	−1	0	8	−1	0	8	−1	0	8	−1	0
9	0.6716	0.7410	9	−0.7410	0.6716	9	−0.6716	−0.7410	9	0.7410	−0.6716
10	0.1951	−0.9808	10	−0.1951	0.9808	10	0.1951	−0.9808	10	−0.1951	0.9808
11	−0.9415	0.3369	11	0.3369	0.9415	11	0.9415	−0.3369	11	−0.3369	−0.9415
12	0.7071	0.7071	12	0.7071	0.7071	12	0.7071	0.7071	12	0.7071	0.7071
13	0.4276	−0.9040	13	0.9040	0.4276	13	−0.4276	0.9040	13	−0.9040	−0.4276
14	−0.9808	−0.1951	14	0.9808	0.1951	14	−0.9808	−0.1951	14	0.9808	0.1951
15	−0.0491	0.9988	15	0.9988	0.0491	15	0.0491	0.9988	15	−0.9988	−0.0491

**Table 2:** $N_{\mathrm{gen}}$ and $N_{\mathrm{in}}$ for a LoRa system with chirp segmentation.

SF	*M*	$N_{\mathrm{gen}}$	$N_{\mathrm{in}}$
6	64	32	25
7	128	64	49
8	256	128	97
9	512	256	193
10	1024	512	385
11	2048	1024	769
12	4096	2048	1537
All		4064	2049

**Table 3:** Total number of samples ($N_{\mathrm{TX}}$) for a standard LoRa system and a system with chirp segmentation: Length-17 and 81 filters.

SF	Standard	$N_{\mathrm{filt}}=17$	$N_{\mathrm{filt}}=81$
6	128	248	1016
7	256	496	2032
8	512	992	4064
9	1024	1984	8128
10	2048	3968	16,256
11	4096	7936	32,512
12	8192	15,872	65,024
All	16,256	22,496	88,032

**Table 4:** $N_{\mathrm{in}}$ for LoRa systems with various levels of quantization.

SF	*M*	Fractional Bit Precision (*B*)				
		2	4	6	8	10
6	64	5	17	23	24	25
7	128	5	17	40	47	48
8	256	5	17	58	89	95
9	512	5	17	65	160	185
10	1024	5	17	65	233	352
11	2048	5	17	65	257	637
12	4096	5	17	65	257	929
All		5	17	65	257	1025

**Table 5:** Total number of samples ($N_{\mathrm{TX}}$) for a system with a length-17 filter, chirp segmentation, and quantization.

SF	Number of Fraction Bits (*B*)				
	2	4	6	8	10
6	68	176	230	239	248
7	100	208	415	478	487
8	164	272	641	920	974
9	292	400	832	1687	1912
10	548	656	1088	2600	3671
11	1060	1168	1600	3328	6748
12	2084	2192	2624	4352	10,400
All	4100	4208	4640	6368	13,280

**Table 6:** Total number of samples ($N_{\mathrm{TX}}$) for a system with a length-81 filter, chirp segmentation, and quantization.

SF	Number of Fraction Bits (*B*)				
	2	4	6	8	10
6	196	688	934	975	1016
7	228	720	1663	1950	1991
8	292	784	2465	3736	3982
9	420	912	2880	6775	7800
10	676	1168	3136	10,024	14,903
11	1188	1680	3648	11,520	27,100
12	2212	2704	4672	12,544	40,096
All	4228	4720	6688	14,560	46,048

**Table 7:** Spectrum analyzer’s settings by specified LoRa bandwidth.

LoRa bandwidth (BW)	125 kHz	250 kHz	500 kHz
Frequency span	500 kHz	1 MHz	2 MHz
Resolution bandwidth (RBW)	5.1 kHz	10 kHz	15 kHz
Video bandwidth (VBW)	16 kHz	30 kHz	47 kHz

**Table 8:** Measured occupied bandwidth (OBW) (kHz) for filtered and quantized LoRa signals with SF=10, varying BW, and varying *B*.

*B*	BW125	BW250	BW500
N/A	130.09	259.99	515.60
10	130.09	259.99	515.60
8	130.09	259.99	515.60
6	130.09	259.99	515.60
4	130.07	259.95	515.58
2	130.04	259.89	515.47

## Abbreviations

The following abbreviations are used in this manuscript:

AWGN	Additive white Gaussian noise
BER	Bit-error rate
CSS	Chirp spread spectrum
FFT	Fast Fourier transform
IoT	Internet-of-things
LUT	Look-up table
OBW	Occupied bandwidth
SNR	Signal-to-noise ratio
SRRC	Square-root raised cosine
